# Effect of fire needle therapy on mild-moderate benign prostatic hyperplasia: Protocol for a randomized controlled pilot trial

**DOI:** 10.1097/MD.0000000000020376

**Published:** 2020-05-22

**Authors:** Tao Zhang, Yun-Qing Xun, Bin Li, Gui-Ling Wang, Lin-Peng Wang, Lian-Cheng Jia, Wei-Guang Li, Xue-Mei Liu, Hui-Lin Liu, Jing-Qing Sun

**Affiliations:** a Department of Acupuncture and Moxibustion, Beijing Hospital of Traditional Chinese Medicine, Capital Medical University, Beijing Key Laboratory of Acupuncture Neuromodulation; b Graduate School, Beijing University of Chinese Medicine; c Department of Urology; d Ultrasound Diagnostics Division, Beijing Hospital of Traditional Chinese Medicine, Capital Medical University, Beijing, China.

**Keywords:** Benign prostatic hyperplasia, fire needle, randomized controlled trial, study protocol

## Abstract

**Background:**

Benign prostatic hyperplasia (BPH) is the most common non-cancerous disease of the prostate and leads to lower urinary tract symptoms in middle-aged and elderly males. Fire needle therapy could improve the lower urinary tract symptoms associated with mild-moderate BPH in clinical practice. The aim of the present pilot study is to assess the preliminary effects of fire needle therapy on mild-moderate BPH.

**Methods:**

The present study is a prospective parallel randomized controlled pilot trial. A total of 60 eligible participants will be randomly assigned to a treatment or control group at a 1:1 ratio. The treatment group will receive fire needle therapy and the control group will receive watchful waiting with lifestyle advice and education regarding BPH. Participants will receive intervention for 4 weeks, with a follow-up period of 4 additional weeks. Adverse events will be recorded to assess the safety and tolerability of fire needle therapy for mild-moderate BPH. The primary outcome will be the change in the International Prostate Symptom Score. The secondary outcomes will include the change in the mean number of nightly urinations, the maximum urinary flow rate, the average flow rate, and the prostate volume as measured by a B-mode ultrasound device. All outcome measures will be observed at baseline and at 4 and 8 weeks following the beginning of treatment.

**Discussion:**

The present study will provide evidence of the preliminary effects of fire needle therapy on mild-moderate BPH and indicate an optimal sample size for future studies.

## 1 Introduction

Benign prostatic hyperplasia (BPH) is a non-cancerous enlargement of the prostate gland, commonly encountered in middle-aged and elderly males.^[1]^ The main manifestation is an enlarged prostate and/or lower urinary tract symptoms (LUTS) without other known causes.^[2]^ LUTS is characterized by storage symptoms, such as urinary frequency, urgency, nocturia, and incontinence, in addition to voiding symptoms, such as hesitancy, progressive dysuria, and intermittent flow, and post-micturition symptoms, such as the feeling of incomplete emptying and post-micturition dribble.^[3]^ There is a positive correlation between the occurrence of BPH and age, and it often arises in males over the age of 40 years old.^[4]^ Studies have indicated that the prevalence of BPH is approximately 25% to 40% in men in their 50 s, about 50% in their 60 s, and more than 75% in their 80 s.^[4–7]^ Of the men who suffer from BPH, up to 50% experience LUTS.^[8]^ The prevalence of BPH in China is similar to that in western countries.^[9]^ Among Chinese urological outpatients, approximately 47.0% are men aged ≥60 with BPH.^[10]^ Studies have shown that Asians with BPH are more prone to suffer from moderate-severe LUTS than their American counterparts.^[11]^ Interventions for BPH include conservative (watchful waiting, behavioral and dietary modifications), pharmacological, and surgical treatments.^[12]^ Although BPH is not considered a risk factor for prostate cancer, it greatly influences patients’ health and quality of life and increases healthcare costs.

Acupuncture has a history of more than 2000 years in China and plays an important role in traditional Chinese medicine.^[13]^ The World Health Organization has composed a provisional list of diseases that could potentially be treated with acupuncture, 1 of which is prostatic disease.^[14,15]^ Recent systematic reviews have suggested that acupuncture has statistically significant beneficial effects on the International Prostate Symptom Score (IPSS) and maximum urinary flow rate (Q_max_) in moderate-severe BPH.^[16,17]^ Fire needle therapy was first recorded in Huangdi Neijing (The Inner Canon of Huangdi), 1 of the earliest medical books in China.^[18]^ It is an acupuncture technique that involves rapid insertion of a red-hot needle into the lesion or acupuncture point to eliminate disease symptoms.^[19]^ Outpatient fire needle therapy is widely used in the treatment of BPH and LUTS; however, high-quality clinical evidence is not yet available, especially that obtained using evidence-based design. Therefore, we designed this randomized controlled pilot trial to assess the preliminary effects of fire needle therapy on mild-moderate BPH and indicate an optimal sample size for future studies.

## 2 Methods

### 2.1 Study design

**Figure 1:**
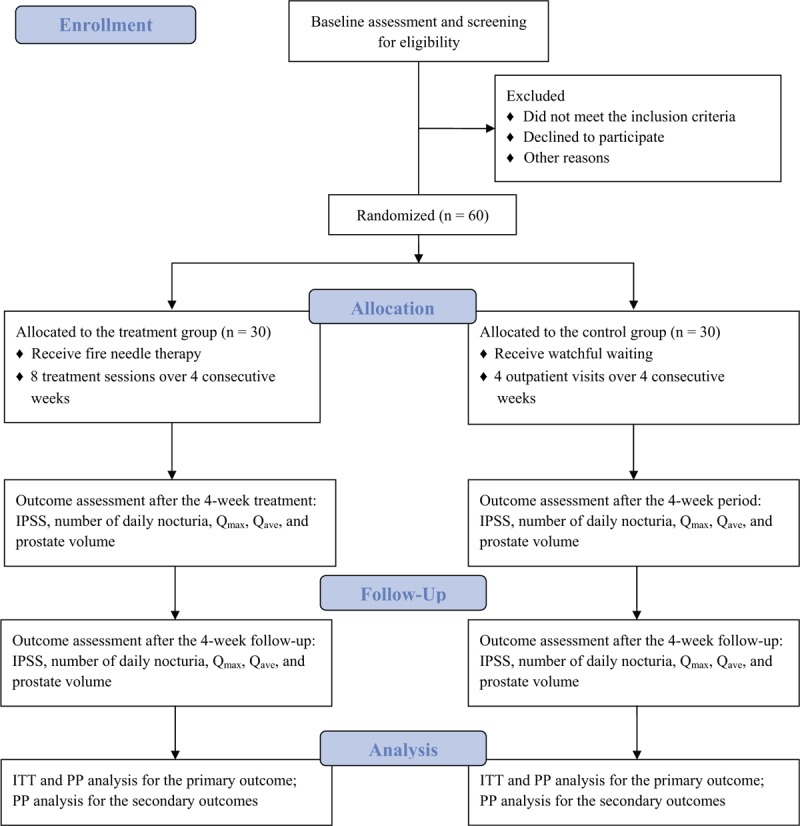
Consolidated standards of reporting trials (CONSORT) flow chart of the present study. IPSS = International Prostate Symptom Score, ITT = intention-to-treat, PP = per-protocol, Q_ave_ = average flow rate, Q_max_ = maximum urinary flow rate.

This is a prospective parallel randomized controlled pilot trial comparing fire needle therapy with watchful waiting. From July 1, 2020 to December 31, 2021, we will recruit patients suffering from mild-moderate BPH according to predefined inclusion and exclusion criteria. Participants will be randomly allocated to a treatment (fire needle therapy) or control (watchful waiting) group and receive treatment for 4 weeks. A 4-week follow-up will take place after the treatment period. All outcomes will be assessed at baseline and at 4 and 8 weeks following the beginning of treatment (Fig. 1).

### 2.2 Sample size

Since there are no previous randomized controlled trials investigating this topic, the present study will be a pilot trial to provide effect size data for sample size calculations in subsequent large-scale randomized controlled trials. The sample size was not calculated based on a power calculation;^[20]^ instead, it was determined from estimates of the number of patients expected to participate and the minimum number of patients needed for an evaluation of the pragmatic purpose of the trial. Therefore, a total number of 60 participants, a sample size of 30 per group, will be recruited during the study, which is larger than the minimum number recommended for pilot studies.^[21]^

### 2.3 Recruitment and baseline assessment

Researchers will recruit participants from outpatient clinics at the Department of Acupuncture and Moxibustion, Beijing Hospital of Traditional Chinese Medicine, Capital Medical University, Beijing, China. Recruitment information will be posted in the hospital, containing a brief introduction of the study, participants, interventions, and contact details, which will be pre-approved by the Institutional Review Board.

Patients who are interested in participating in the study will be provided with detailed written and verbal information, and those who volunteer to enroll will be asked to complete an IPSS questionnaire and a 2-week nocturia record and undergo a routine urine test, prostate ultrasound, and uroflowmetry test as a baseline assessment. The results will be used to decide whether to include the patients in the study according to the inclusion and exclusion criteria. All recruited participants will give their written informed consent with a witness present prior to the beginning of the study, and subsequently complete a general information form, including name, gender, age, and medical history. All participants will be treated respectfully according to the Declaration of Helsinki and will be able to withdraw from the study at any time for any reason. Personal information regarding the participants will be collected and kept confidential.

### 2.4 Inclusion criteria

Patients are required to meet the following criteria:

(1) aged 40 to 80 years old;
(2) diagnosed with BPH more than 3 months previously;
(3) have a syndrome differentiation in traditional Chinese medicine conforming to “kidney yang deficiency;”^[22]^
(4) have abstained from medication for BPH for longer than 6 months prior to the start of treatment;
(5) have an IPSS score < 19 (mild-moderate BPH);
(6) have a Q_max_ < 15 mL/s or a average flow rate  < 10 mL/s;
(7) have stable life signs; and
(8) participate voluntarily in the study and sign informed consent prior to treatment.

### 2.5 Exclusion criteria

Patients with any of the following conditions will be excluded:

(1) oliguria and anuria caused by bladder or prostate malignancy, urolithiasis, or acute/chronic renal failure;
(2) urinary dysfunction caused by neurogenic bladder, bladder neck fibrosis, or urethral stricture;
(3) gonorrhea and urinary tract infection;
(4) failure of invasive therapy for BPH;
(5) local organ, muscle, or nerve injury caused by pelvic surgery or trauma;
(6) abdominal aneurysms, hepatosplenomegaly, and serious cerebrovascular, cardiovascular, respiratory, digestive, hematological, or psychiatric diseases;
(7) blood coagulation disorders or patients requiring administration of anticoagulant medication, such as warfarin or dabigatran;
(8) cognitive dysfunction, psychosis, or patients who are unable to cooperate with the examination and treatment.

### 2.6 Randomization and allocation concealment

Participants will be randomly assigned to 2 groups at a 1:1 ratio. The generation and allocation of a random sequence for group assignment will be conducted using the Statistical Analysis System (V.9.1.3; SAS Institute Inc., Cary, North Carolina) by the Research Center of Clinical Epidemiology, Beijing Hospital of Traditional Chinese Medicine, Capital Medical University, Beijing, China. Researchers involved in randomization will be blinded and will not take part in the recruitment and inclusion of participants. Opaque computer-generated sealed envelopes will be produced to conceal allocation. The envelopes will be numbered consecutively with a serial number on the outside and will contain the allocation information inside. An envelope will be opened as a participant is enrolled in the study following completion of the baseline assessments and written informed consent. Participants will be allocated to 1 of the 2 groups and receive the related intervention. The random allocation sequence and opaque sealed envelopes will be kept separately by 2 specific researchers.

### 2.7 Blinding

The outcome assessors, data managers, and statisticians will remain blind to the participants’ group assignment. Acupuncturists, outcome assessors, data managers, and statisticians will not be allowed to communicate with each other regarding the participants’ allocation.

### 2.8 Interventions

#### 2.8.1 Treatment group

Participants in the treatment group will receive fire needle therapy in the outpatient clinics of the Department of Acupuncture and Moxibustion at the hospital. Interventions will be performed in accordance with the STRICTA.^[23]^ All acupuncturists will be registered practitioners of traditional Chinese medicine with at least 5 years of clinical experience. The fire needle treatments and manipulations will be standardized among acupuncturists using specific training. Fire needles (0.65 × 45 mm; Beijing Zhongyan Taihe Medical Instrument Co., Ltd., Beijing, China) will be used. Other medication for BPH will be prohibited during the trial.

The following acupoints will be used: CV4 (*guanyuan*), CV2 (*qugu*), and bilateral ST28 (*shuidao*). All acupoints will be located according to the World Health Organization Standardized Acupuncture Points Location.^[24]^ The manipulation of fire needle therapy will be conducted according to Chinese national standard Standardized Manipulations of Acupuncture and Moxibustion.^[19]^ During treatment, participants will maintain a supine position after urination. The acupoints will be sterilized twice, from the center to the outside of an imaginary circle with a diameter of 5 cm, using 75% alcohol, or 0.5% to 1% iodophor if an alcohol allergy is present. After the skin is dry, the tip and middle of the fire needle will be heated until it turns red, following which the acupoint will be directly penetrated by the needle to a depth of 25 mm. The needle will be subsequently withdrawn without retention after the therapeutic depth has been reached, and the puncture wound will be covered with a sterile cotton ball to avoid bleeding.

Treatment will be conducted twice per week. Participants will be treated once a day and will receive a total of 8 treatment sessions over 4 consecutive weeks.

#### 2.8.2 Control group

Participants in the control group will receive watchful waiting, which is 1 of the conventional treatments suggested by both the Chinese and EAU Guidelines on the Management of Non-neurogenic Male LUTS.^[10,25]^ Watchful waiting and behavioral and dietary modifications are recommended as initial treatments for mild-moderate BPH (IPSS score < 19) in patients with non-bothersome LUTS. Accordingly, participants will receive education regarding BPH, reassurance regarding prognosis, and lifestyle advice once per week. Participants will receive a total of 4 outpatient visits over 4 consecutive weeks. Medication for BPH will be prohibited during the trial.

#### 2.8.3 Follow-up

After the 4-week treatment, follow-up will be conducted among all participants for 4 weeks. Fire needle therapy and BPH-related treatment are not permitted during follow-up.

### 2.9 Outcome measures

**Figure 2:**
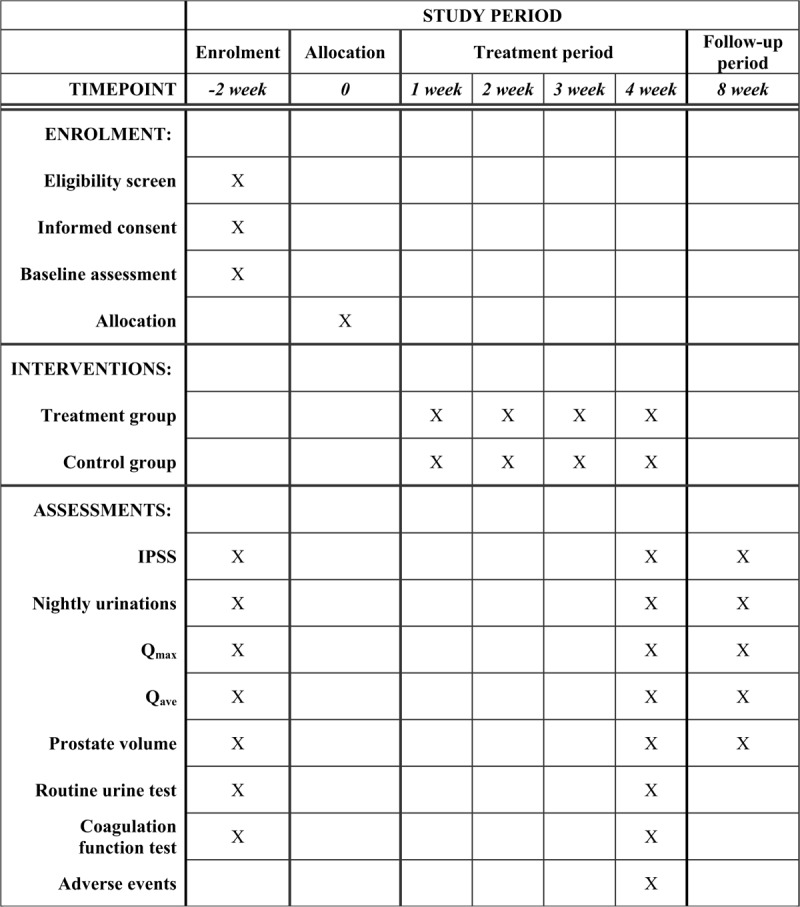
Schedule of enrolment, interventions, and assessments. IPSS = International Prostate Symptom Score, Q_ave_ = average flow rate, Q_max_ = maximum urinary flow rate.

The primary outcome is the change in IPSS over the 2-week baseline, 4-week treatment, and 4-week follow-up periods. The secondary outcomes are the changes in the mean number of nightly urinations, Q_max_ and average flow rate, and prostate volume as measured by a B-mode ultrasound device over the 2-week baseline, 4-week treatment, and 4-week follow-up periods. All outcome measures will be observed at baseline and 4 and 8 weeks following the beginning of treatment (Fig. 2).

### 2.10 Adverse events

Adverse events will be recorded to access the safety of fire needle therapy. The common and expected adverse events include local hematomas, inflammation, bending of the needle, fainting, unbearable prickling, discomfort persisting for longer than 1 hour after acupuncture, local infections, abscesses, and local pruritus. All participants will receive a routine urine test and coagulation function test at baseline and at the end of the 4-week treatment period. Researchers will evaluate the relationship between adverse events and the interventions. All adverse events will be reported to the primary investigator and the Research Ethics Committee of Beijing Hospital of Traditional Chinese Medicine to decide whether the participants need to reveal allocated intervention and withdraw from the trial. The participants who experience adverse events will be treated with the relevant conventional therapy or hospitalization if necessary.

### 2.11 Data management and monitoring

Researchers who manage data will be trained prior to enrollment. Assessors will be in charge of the acquisition of participant information and outcome assessment during the study. Data collectors will transfer the data from the case reports to electronic form. All paper and electronic data will be kept safely in the office of the Department of Acupuncture and Moxibustion. Only investigators of our study team can have access to the final trial data. Others getting written requests from the project administration can be allowed. The study will be regularly monitored and audited by the Data Management and Monitoring Committee of the Good Clinical Practice Department of Beijing Hospital of Traditional Chinese Medicine, Capital Medical University, Beijing, China. The committee is independent of the study researchers and has no competing interests; it will examine original case report forms, monitor the quality and completeness of the data, verify the recording of adverse events, and ensure that the study complies with the principles of the protocol. The periodic review is done every 3 months.

### 2.12 Statistical analysis

The SPSS statistical package (version 20.0, International Business Machines Corporation, China) will be used to analyze the data. The primary outcome will be analyzed by both intention-to-treat and per-protocol analysis. The data missing from intention-to-treat analysis will be replaced with the last measured value. The secondary outcomes will be analyzed by per-protocol analysis. Categorical variables will be displayed as percentages. Continuous variables will be presented as the mean ± standard deviation if the data fit a normal distribution in 2 groups. Otherwise, the data will be presented as the median (interquartile range). The Kolmogorov–Smirnov test will be applied as the normal distribution test for continuous variables. The *t*-test or Wilcoxon test will be used to compare the difference between groups according to the results of the Kolmogorov–Smirnov test. The results for categorical data will be compared using the chi-squared test. The statistical significance level will be set at 0.05 (2-sided), with a 95% confidence interval.

### 2.13 Withdrawal and dropout

If participants use a combination of treatments that are prohibited by the study protocol, withdraw their consent, or cease communication, they will be excluded from the study. Researchers will report the reasons for withdrawal and dropout and acquire the time of last treatment and outcome measures where possible.

### 2.14 Ethics and dissemination

The study protocol version is V2.0, dated March 3, 2020. It follows the principles of the Declaration of Helsinki and was approved by the Research Ethics Committee of Beijing Hospital of Traditional Chinese Medicine, Capital Medical University, Beijing, China on 25^th^ March 2020 (approval No. 2020BL02-004-02). All recruited participants will give their written informed consents, which includes consent for blood and urine tests. All blood and urine samples taken from participants will be destroyed after the tests. Participants may withdraw consent or cease to participate at any time for any reason. The present study has been registered with the Chinese Clinical Trial Registry (ChiCTR; website: http://www.chictr.org.cn) as a current controlled trial (Identifier: ChiCTR2000031841). The results will be disseminated through peer-reviewed publications, a medical Master's thesis, or conference presentations. The data will be anonymized prior to publication to protect participants’ privacy.

## 3 Discussion

BPH is a common health problem among middle-aged and elderly men. The number of BPH cases increases each year and it has become 1 of the major health concerns in the male population. The etiology and pathophysiology of BPH are complex; hyperplasia of the prostate gland is multifactorial, involving aging, hormone levels, late activation of cell growth, and genetic and lifestyle elements.^[26]^ These factors cause bladder outlet obstruction and an increase in smooth muscle, which influences the normal urine flow and leads to LUTS.^[25]^ Since it is regarded as a major public health concern among older men, research on the prevention and treatment of BPH has attracted significant attention. BPH treatment including pharmacological therapies, minimally invasive surgery, and open prostatectomy has shown benefits; however, these treatments have limitations such as high cost, limited clinical effect, and hidden costs of the significant risk of serious adverse events. Complementary and alternative medical treatment may become a choice for such chronic diseases in the elderly due to the advantage of relieving clinical symptoms.

Fire needle therapy, with a history of more than 2000 years, is widely used in clinical practice in China. According to the published clinical trials, systematic reviews and meta-analysis, and guidelines and national standards for acupuncture-moxibustion, the indications of fire needle therapy cover a wide range, including cardiovascular, respiratory, digestive, urogenital, orthopedic, rheumatic, neurological, dermatological, and gynecological diseases. Mechanistic research has indicated that fire needle stimulation at lesions or acupoints can improve or even eliminate the pathological changes in local tissues, such as edema, hyperemia, exudation, adhesion, calcification, contracture, and ischemia. Moreover, it can improve local blood circulation, enhance local tissue metabolism, regulate serum cortisol levels, and reduce inflammation by autophagy.^[18]^ Based on these mechanisms, fire needle therapy is commonly used for BPH and LUTS in East Asian countries as manual acupuncture, electro-acupuncture, and moxibustion; however, existing research regarding fire needle therapy for BPH mainly consists of case series studies with small sample sizes, and high quality clinical research is rare. Therefore, we designed this randomized controlled trial using fire needle therapy as an intervention in the treatment group. By applying the function “warming the kidney and tonifying yang” of fire needle therapy in traditional Chinese medicine theory, the present study focuses on BPH patients with “kidney yang deficiency” syndrome differentiation.^[18,22]^

Selection of a control group is another major issue in planning the design of a study. We searched the Cochrane Central Register of Controlled Trials, MEDLINE, EMBASE, the Chinese Biomedical Database, the China National Knowledge Infrastructure, the VIP Database, and the Wanfang Database for clinical trials and systematic reviews describing acupuncture for BPH. As a result, the controlled intervention could be classified into 2 groups: sham acupuncture and medication. The use of sham acupuncture as a control was denied due to the clinical difficulty in performing sham fire needle manipulation. Since there is a lack of evidence supporting the use of fire needle therapy for BPH, we decided to use a blank control. After evaluating several guidelines for BPH and LUTS, we finally selected watchful waiting as the controlled intervention, which is strongly recommended for men with mild-moderate BPH (IPSS score < 19) who are minimally bothered by their symptoms.^[25]^ Should acupuncture prove to be effective in this study, we will subsequently compare fire needle therapy with a known medication as a positive control to clarify its specific effects.

However, the present study still has some unavoidable limitations. Despite the blinding of assessors, data managers, and statisticians, the therapists and participants cannot be blinded due to the nature of fire needle manipulation, which may lead to the occurrence of performance and detection biases. In addition, the 4-week treatment period with 8 treatments and the 4-week follow-up period are not sufficient to evaluate the long-term effects of fire needle therapy on BPH. Future trials should include more frequent treatments and a longer follow-up period to effectively investigate the long-term effects. Moreover, the sample size of this study is small, since it is proposed as a pilot for further studies. The results of the present study will offer preliminary data regarding the safety, tolerability, and effect of fire needle therapy for mild-moderate BPH and provide evidence for the optimal sample size in subsequent large-scale multi-center randomized controlled trials. The series studies of fire needle therapy will be a preliminary investigation into the potential enhancement of a currently used intervention and may contribute to changes in future clinical practice.

## Author contributions

**Conceptualization:** Tao Zhang, Hui-Lin Liu, Jing-Qing Sun.

**Funding acquisition:** Tao Zhang and Jing-Qing Sun.

**Investigation:** Yun-Qing Xun, Xue-Mei Liu, Lian-Cheng Jia.

**Methodology:** Lin-Peng Wang, Bin Li, Hui-Lin Liu.

**Project administration:** Tao Zhang.

**Supervision:** Gui-Ling Wang, Wei-Guang Li.

**Writing – original draft:** Tao Zhang, Yun-Qing Xun.

**Writing – review &editing:** Jing-Qing Sun, Hui-Lin Liu.
